# Functional metagenomics uncovers nitrile-hydrolysing enzymes in a coal metagenome

**DOI:** 10.3389/fmolb.2023.1123902

**Published:** 2023-03-17

**Authors:** Arunmozhi Bharathi Achudhan, Priya Kannan, Lilly M. Saleena

**Affiliations:** Department of Biotechnology, School of Bioengineering, SRM Institute of Science and Technology, Kattankulathur, Tamil Nadu, India

**Keywords:** functional metagenomics, nitrilase, nitriles, artificial intelligence, unclassified microorganisms

## Abstract

**Introduction:** Nitriles are the most toxic compounds that can lead to serious human illness through inhalation and consumption due to environmental pollution. Nitrilases can highly degrade nitriles isolated from the natural ecosystem. In the current study, we focused on the discovery of novel nitrilases from a coal metagenome using *in silico* mining.

**Methods:** Coal metagenomic DNA was isolated and sequenced on the Illumina platform. Quality reads were assembled using MEGAHIT, and statistics were checked using QUAST. Annotation was performed using the automated tool SqueezeMeta. The annotated amino acid sequences were mined for nitrilase from the unclassified organism. Sequence alignment and phylogenetic analyses were carried out using ClustalW and MEGA11. Conserved regions of the amino acid sequences were identified using InterProScan and NCBI-CDD servers. The physicochemical properties of the amino acids were measured using ExPASy’s ProtParam. Furthermore, NetSurfP was used for 2D structure prediction, while AlphaFold2 in Chimera X 1.4 was used for 3D structure prediction. To check the solvation of the predicted protein, a dynamic simulation was conducted on the WebGRO server. Ligands were extracted from the Protein Data Bank (PDB) for molecular docking upon active site prediction using the CASTp server.

**Results and discussion:** *In silico* mining of annotated metagenomic data revealed nitrilase from unclassified *Alphaproteobacteria*. By using the artificial intelligence program AlphaFold2, the 3D structure was predicted with a per-residue confidence statistic score of about 95.8%, and the stability of the predicted model was verified with molecular dynamics for a 100-ns simulation. Molecular docking analysis determined the binding affinity of a novel nitrilase with nitriles. The binding scores produced by the novel nitrilase were approximately similar to those of the other prokaryotic nitrilase crystal structures, with a deviation of ±0.5.

## Introduction

Cyanide-containing compounds are known as nitriles and are widely distributed in the natural environment. They are generated by different plants in various forms, such as ricinine, phenyl acetonitrile, cyanogenic glycosides, and β -cyanoalanine (Sewell et al., 2003). Anthropogenic activities have substantially influenced the production of vast quantities of nitrile compounds. Nitriles are naturally poisonous and are recognised to be a leading cause of environmental pollution, which is detrimental to human health (Li et al., 2013). Most of the cyanide in soil and water comes from effluents that contain a variety of inorganic cyanides and nitriles. Contamination is caused by using herbicides with the nitrile group, such as 2,6-dichlorobenzonitrile and bromoxynil (3,5-dibromo-4-hydroxybenzonitrile). Nitrile pollution can also be caused by the exhaust from cars (Nigam et al., 2017). The majority of nitrile poisoning symptoms include abdominal pain, seizures, breathing problems, sore throat, difficulty falling asleep, and damage to the kidneys (Kupke et al., 2016; Tanii, 2017).

**FIGURE 1:**
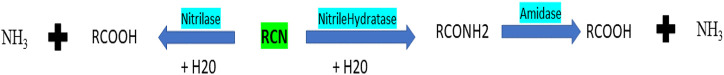
Mechanism of nitrilase, nitrile hydratase, and amidase.

Nitrile compounds can be degraded by using microbes or chemicals. Chemical degradation of nitriles involves harsh reaction conditions and generates excess waste (Wang, 2015). Nitrile-hydrolysing enzymes can convert various nitriles to acids. Enzymes that hydrolyse nitrile include nitrilases (EC 3.5.5.1), nitrile hydratases (EC 4.2.1.84), and amidases (EC 3.5.1.4) (Figure 1). These enzymes are utilised extensively in the production of amides and organic acids, both of which are extremely valuable to the manufacturing industry (Egelkamp et al., 2019).

The benzonitrile analogues chloroxynil, bromoxynil, and ioxynil are efficiently degraded by the soil *Actinobacteria* *Rhodococcus rhodochrous* PA-34*, Rhodococcus* sp*.* NDB 1165, and *Nocardia globerula* NHB-2, and these nitrile degraders should be studied for the bioremediation of benzonitrile herbicide-contaminated soils (Veselá et al., 2010). Nitrilase enzymes are used as good biocatalysts in a wide range of synthetic processes, leading to a huge rise in demand. Hydrolysis of the ricinine nitrile group was first discovered in the soil-isolated bacterial strain belonging to the genus *Pseudomonas.* Many nitrilases have been purified, characterised, immobilised, gene cloned, overexpressed in host strains, and used in industrial plants (Amrutha and Nampoothiri, 2022; Gong et al., 2012).

Advanced bioinformatics tools and techniques are used more often than traditional methods to find or screen a new nitrilase gene. This helps in identifying the protein’s suitable substrates (Jones et al., 2020; Klasberg et al., 2016). The maximum synthesis of propionic acids, which have applications in the food and chemical industries, was demonstrated by nitrilase from *Bacillus sp.* (BITSN007) in a study on the biotransformation of nitrile compounds to valuable acids (Biocatalysis of Different Nitriles to Valuable Acids, 2021). The Tyr141Ala mutation in the nitrilase from *P. fluorescens* EBC191 led to a nitrilase variant that can convert aromatic and aliphatic substrates (Brunner et al., 2018). There is a need for new nitrile-degrading enzymes, particularly those with the wide substrate-catalysing properties required for environmental remediation. The study employs shotgun sequencing of lignite samples collected from the coal mine. After extracting the coal metagenomic data, we identified an unclassified bacterium that codes for the nitrilase enzyme. The primary amino acid sequence was searched for conserved regions and domain findings. The secondary and tertiary structures were also identified for the nitrilase protein, with different types of nitriles (substrates) used to analyse their binding efficiency by molecular docking analysis. Binding energy was also calculated for other reference prokaryotic crystal structures and compared to the predicted structure. This study focuses on identifying nitrilase enzymes from metagenomic data and exploring their binding affinity with a wide range of nitriles.

## Materials and methods

### Sample isolation and sequencing

A lignite sample from the coal mine in Neyveli, India (11°35′34.44″N and 79°29′29.04″E), was collected. The metagenomic DNA was isolated from the lignite sample using the PowerMax soil DNeasy kit (QIAGEN). The isolated metagenomic DNA was then subjected to shotgun sequencing on an Illumina HiSeqTM 2000 platform to generate paired-end sequences.

### Metagenomics data analysis

The forward and reverse end reads in the FASTQ format were used as the input in the FASTQC tool (Andrews, 2010). The generated output HTML files were merged using MultiQC (Ewels et al., 2016) to create a single HTML file report containing the quality statistics of the reads. The forward and reverse FASTQ files are the input for the MEGAHIT assembler (Li et al., 2015). A k-mer value of 99 and a minimum contig length of 200 parameters were assigned. The output was generated as contigs in a single FASTA file. The obtained contigs were analysed in the QUAST tool (Gurevich et al., 2013) for the number and size of the contigs.

### Taxonomical and functional findings

The contigs in FASTA format were annotated using the SqueezeMeta tool, an automated pipeline (Tamames and Puente-Sánchez, 2019). First, protein-coding genes were predicted from the contigs using Prodigal, and amino acid and nucleotide sequences were generated in the FASTA files. The results of these annotated nucleotide sequences were automatically loaded as input into Diamond, which searched the GenBank nr database for taxonomical classification and the KEGG database for functional annotation. The term “Nitrilase” was searched using the grep script in tab-separated value files. KEGG IDs and contig IDs were noted for identifying taxonomy and extracting the nitrilase nucleotide and amino acid sequences.

### Sequence alignment and phylogenetic analysis

The identified amino acid sequence in FASTA format was uploaded in BlastP (Johnson et al., 2008) and ran against the NCBI database of protein reference sequences to find similar sequences. Similar sequences were chosen based on the >50% identity of the matches with the query sequence. These sequences above the threshold were downloaded in FASTA format in a single file along with the query sequence. This FASTA file was uploaded using MEGA 11 software (Tamura et al., 2021) and was aligned using Clustal Omega. The evolutionary history of these sequences was created using the neighbour-joining method by selecting the phylogeny tab in the software application.

### Conserved region analysis

The amino acid sequence in FASTA format was submitted with the default parameters in the NCBI–CDD (Marchler-Bauer et al., 2017) and InterProScan (Quevillon et al., 2005) databases to predict the homologous superfamily, conserved domain, conserved region, Gene Ontology, and NCBI-CDD from the query sequence of amino acids.

### Physiochemical properties of nitrilase enzymes

The amino acid sequence was pasted in FASTA format into a query box of the ExPASy’s ProtParam (Gasteiger et al., 2003) server and submitted to identify the physical and chemical properties of the protein sequence. The server page quantifies the number of amino acids, the molecular weight, the number of negatively charged residues, the instability index, the theoretical pI, and the grand average of hydropathicity.

### Structure prediction

The secondary structure was predicted by uploading an amino acid sequence in FASTA format to the NetSurfP tool with the default parameters (Høie et al., 2022). The amino acid sequences were used in the software package Chimera X version 1.4 (Pettersen et al., 2021; Goddard et al., 2018) to perform the AlphaFold2 tool for 3D structure prediction. This was predicted in the software programme by choosing the AlphaFold2 option from the Structure Prediction tab under Tools. The amino acid sequence was uploaded in the query box and submitted with the default parameters. The predicted tertiary structure was uploaded into the PROCHECK tool (Laskowski et al., 1993) to create the Ramachandran plot, which checks to validate the stereochemical quality of the protein structure.

### Molecular dynamic simulation

The predicted structure was submitted for molecular dynamics simulation on the WebGRO server (Bekker et al., 1993) to check its stability. Using the GROMOS96 43a1 force field settings, the complex system was solvated using a simple point charge (SPC) water model in a triclinic periodic box. The complex system was maintained at a salt concentration of 0.15 M by adding a suitable amount of Na^+^ and Cl^−^ counterions. Using the steepest descent approach, energy reduction was achieved in 5,000 steps. Constant amount, volume temperature (NVT/NPT), and pressure equilibrium types were used. The temperature was set to 300 K, and the pressure was set to 1.0 bar. The simulation time was 100 ns and was conducted with 1,000 frames per simulation. Finally, the simulation result was analysed based on the time-dependent root mean square deviation (RMSD) of the given structure and the root mean square fluctuation (RMSF) of each residue.

### Protein and ligand preparation

The homepage of the Protein Data Bank (PDB) (Burley et al., 2017) was searched for the 3D nitrilase protein structure. X-ray diffraction was selected using the filter option, and the crystal structure of nitrilase proteins was retrieved in PDB format. Nitriles were downloaded from the PubChem database (Kim et al., 2020) in SDF format. The ligands were converted to the PDB format using PyMOL (Schrödinger, 2000), and protein and ligand formats were changed to the PDBQT format for molecular docking using the AutoDockTools-1.5.7 tool.

### Active site predictions

The protein structures’ active site residues were predicted by uploading the PDB files of nitrilase proteins in the computed atlas of the protein surface topography −3.0 (CASTp) server (Tian et al., 2018). The alpha shape theory’s pocket algorithm calculated the active pockets or binding sites, with large pockets with high volumes likely to contain enzyme-binding sites for the interaction between proteins and ligands.

### Molecular docking analysis

In AutoDockTools-1.5.7, the protein structures in the PDB format were used as input to create a grid file by placing a grid box in the protein’s predicted active site for the binding of ligand molecules. The inputs of a protein, a ligand in PDBQT format, and a grid file in text format were used to perform a molecular docking analysis in AutoDock Vina (Eberhardt et al., 2021) with the default parameters. The output was the protein–ligand complex in PDB format. The complex in PDB format was used to study the interaction of amino acids with ligands using the LigPlot + tool (Laskowski and Swindells, 2011).

## Results

### Metagenomic data analysis

FASTQC and MULTIQC determined the paired-end reads to be within the Phred score value of 36, considering that the raw reads to be of standard quality (Bin Kwong et al., 2017). The quality reads of 150-bp length from 32 GB of data were processed for assembly. The statistics of the assembled contigs were evaluated using the QUAST tool, resulting in 226 contigs with >50,000 bp, 1,240 contigs with >25,000 bp, 5,265 contigs with >10,000 bp, 11,596 contigs with >5000 bp, and 73,144 contigs with >1000 bp.

### Taxonomical and functional findings

The phyla observed in significant numbers belong to *Proteobacteria* (76%), *Actinobacteria* (8%), *Firmicutes* (8%), *Spirochaetes* (2%), *Bacteroidetes* (1.5%), *Chloroflexi* (0.8%), *Planctomycetes* (0.5%), *Cyanobacteria* (0.2%), [Thermi] (0.1%), *Fusobacteria* (0.1%), and *Acidobacteria* (0.1%). Using “grep,” the results from SqueezeMeta were searched for nitrilase, and then organisms involved in nitrilase enzymes were identified. In total, 27 organisms were identified, of which four were from unclassified microorganisms and belonged to the KEGG ID K01501 with the metabolic pathway number EC:3.5.5.1. For the novel protein approach, long amino acid sequences and unclassified microorganisms were preferred. A single amino acid sequence of 261 base pairs encoding an unclassified *Alphaproteobacteria* was selected*.*

### Sequence alignment and phylogenetic analysis

**FIGURE 2:**
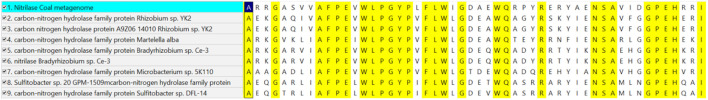
Multiple sequence alignment of the selected eight sequences with the identified novel nitrilase sequence. The highlighted residues are the conserved amino acid sequences. The identified nitrilase sequence from the coal metagenome showed similarity to the 39th site of the other selected protein sequences.

**FIGURE 3:**
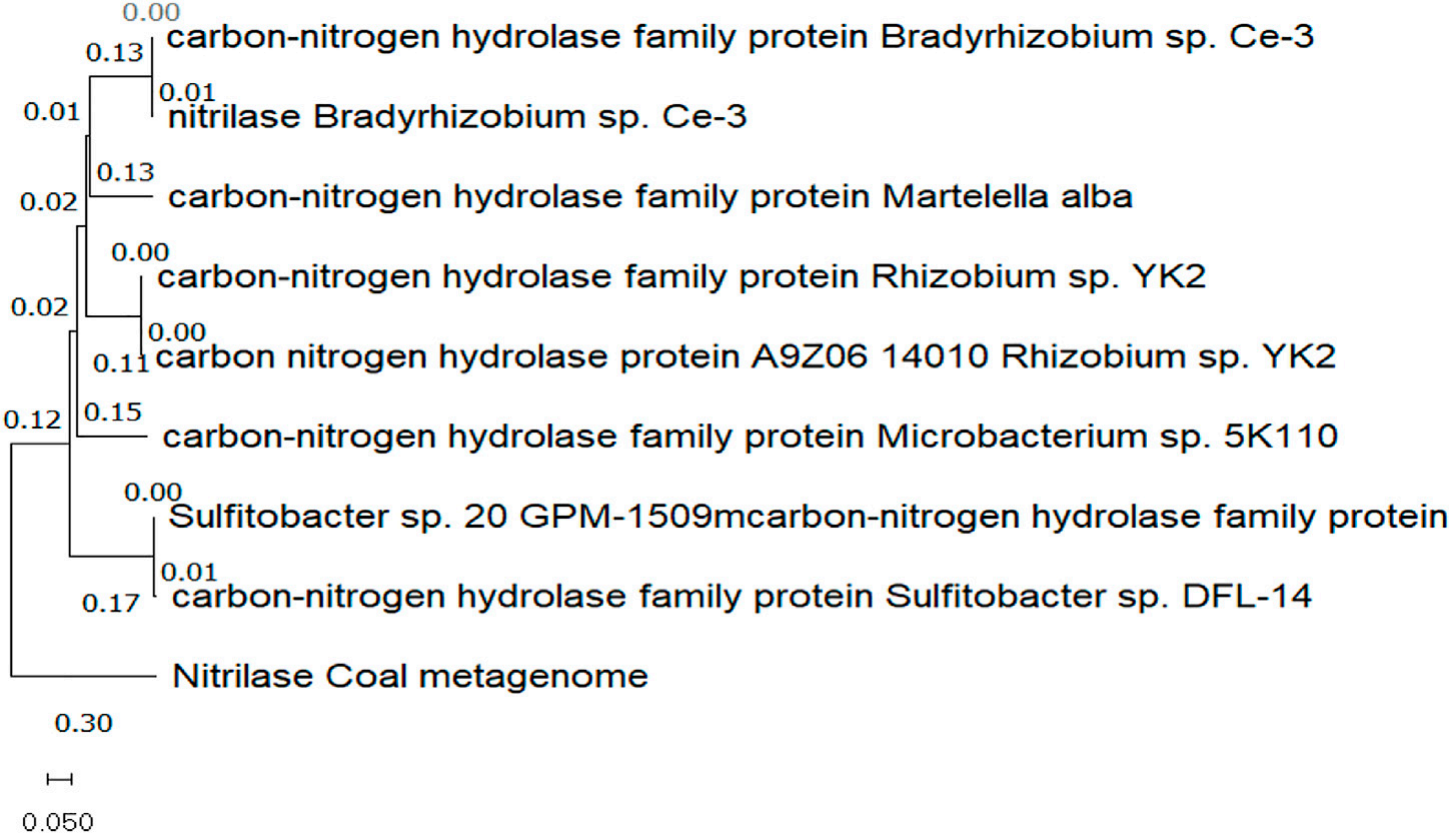
Phylogenetic tree showing the evolutionary relationship between nitrilase from the coal metagenome and other eight selected protein sequences from the NCBI database.

Using BlastP, the target protein sequence was compared to the NCBI database of protein reference sequences. Eight sequences above the threshold were downloaded from NCBI-BLAST, and a phylogenetic tree was built along with the identified nitrilase sequence using the MEGA11 tool. Figure 2 shows the multiple sequence alignment of all the sequences. The branch lengths marked next to the branches on the tree are shown to scale and are in the same units as the evolutionary distances, which are used to estimate the phylogenetic tree (Figure 3). The scale value of 0.050 shows the genetic change between the protein sequences. The branch of the nitrilase coal metagenome is the longest, with a length of 0.30, and is therefore known to have greater genetic change than other protein sequences. The software application calculated evolutionary distances using the Poisson correction method and amino acid substitutions per site. This analysis involved nine protein sequences. There were a total of 309 positions in the final data set, and 139 residues were conserved among the protein sequences.

### Conserved region and domain findings

According to the analysis of the InterProScan database and NCBI-CDD results, a carbon–nitrogen hydrolase domain was found in the 4–19 amino acid position in the protein sequence, indicating it to be a member of the nitrilase superfamily. InterProScan classifies three domains: cellular component, molecular function, and biological process. The predicted biological process for the identified protein is involved in the metabolic process of the nitrogen compounds (GO: 0006807), and the molecular function is involved in catalytic activity (GO:0003824). Based on the unclassification, amino acid sequence length, conserved region, and domain analysis, it is considered to be a novel nitrilase enzyme.

### Physiochemical characteristics of amino acid sequences

**FIGURE 4:**
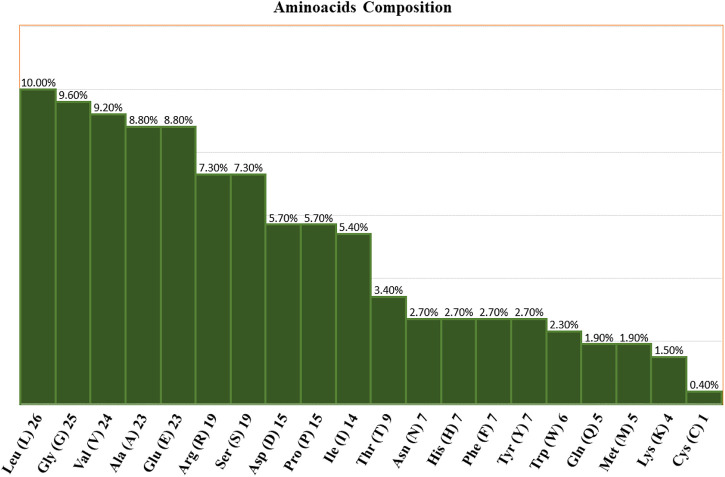
Amino acid compositions of the nitrilase enzyme.

Using the ProtParam tool, the physiochemical characteristics of the novel protein sequence were calculated. The compositions of the amino acids are shown in Figure 4. The result shows that the negatively charged aspartic acid and glutamic acid were higher than the positively charged arginine and lysine. The protein has an acidic nature because of its 4.86 isoelectric points. The total number of atoms is around 4,010, including 1,275 carbon, 1,990 hydrogen, 354 nitrogen, 385 oxygen, and 6 sulphur atoms. Proteins with a GRAVY score below zero are hydrophilic, while proteins with a GRAVY score above zero are hydrophobic. The novel protein has a GRAVY value of −0.126, which indicates that the protein is inherently hydrophilic. The protein has an instability index of 53.02, which indicates that it is unstable because a protein with an instability index of 40 or less was stable in the test tube. The sequence of amino acids was also used to estimate the protein’s half-life. For yeast cells, the half-life period is > 20 h (*in vivo*), and for *E. coli*, the half-life period is >10 h (*in vivo*) (Gasteiger et al., 2005).

### Structure predictions

**FIGURE 5:**
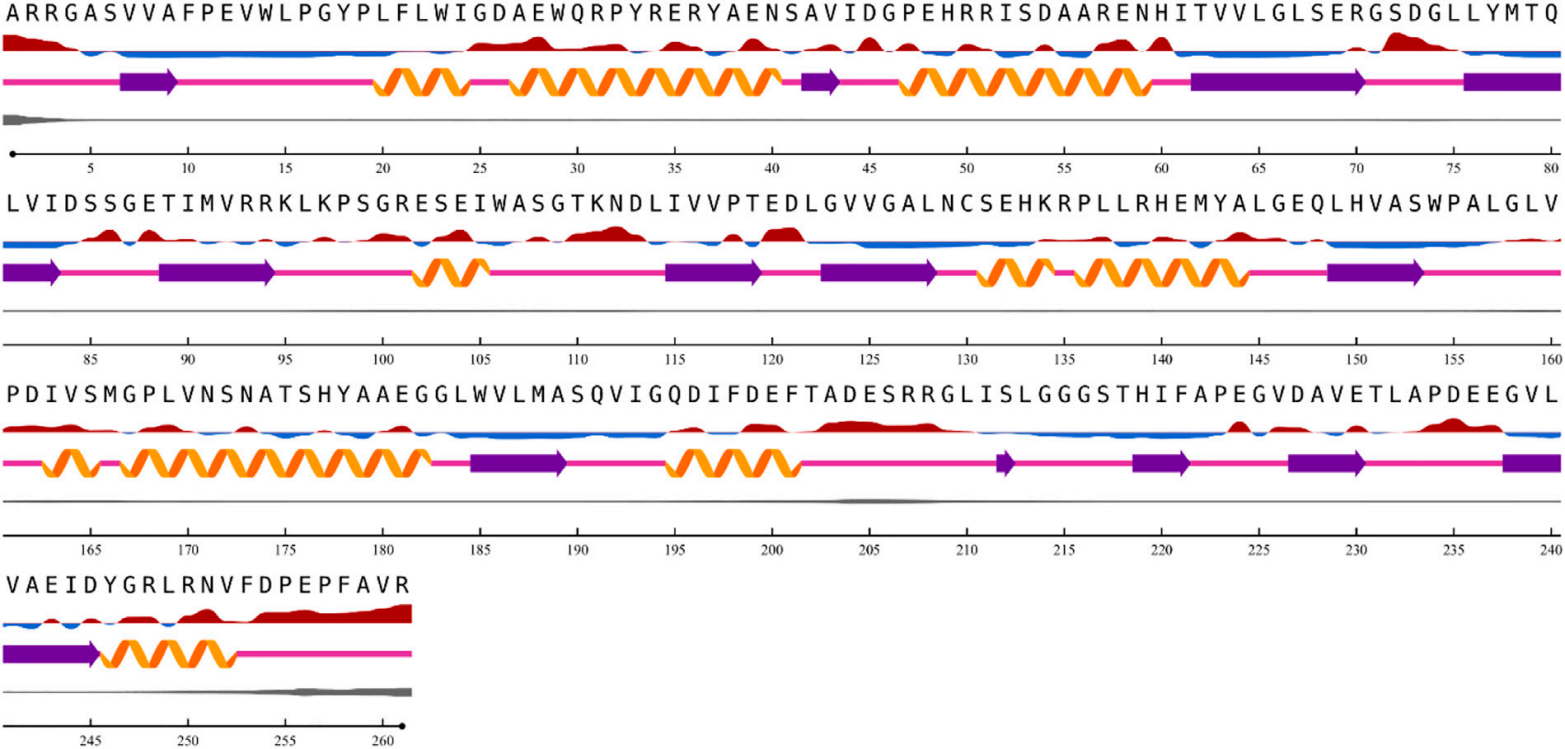
Prediction of nitrilase secondary structure.

The results from the NetSurfP version 2.0 server revealed that the nitrilase protein has 10 helices, 16 strands, and 23 coils in its secondary structure (Figure 5).

**FIGURE 6:**
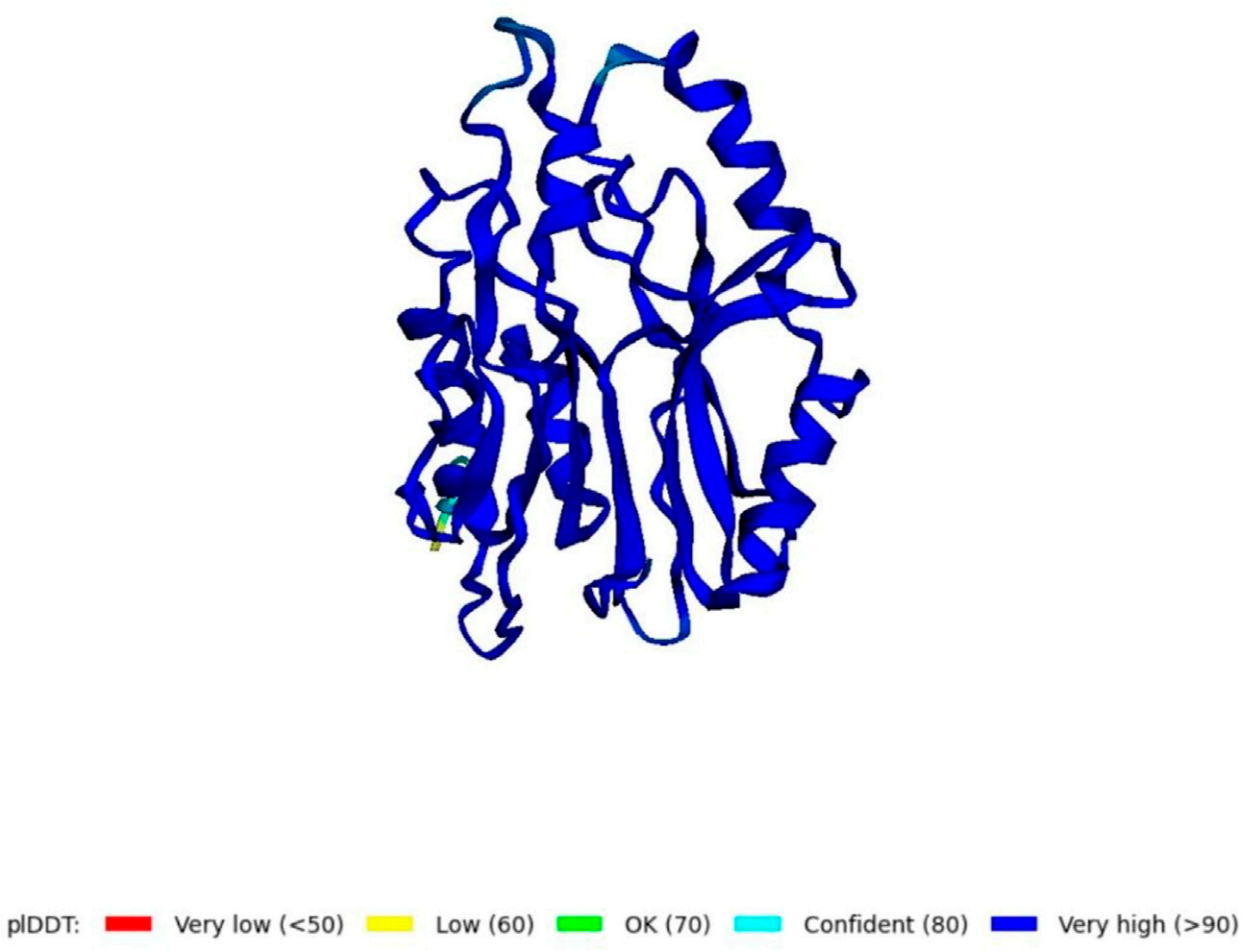
Prediction of a novel nitrilase structure using AlphaFold2.

Using the amino acid sequence with the default parameters, the nitrilase protein structure was predicted in AlphaFold2 (Figure 6). In AlphaFold2, the predicted local distance difference test (pLDDT) calculates the mean confidence value ranging from 0 to 100. Here, the predicted nitrilase’s mean pLDDT score was 95.8. A mean confidence value of 90 and above is said to agree with an experimental structure (Jumper et al., 2021). The Ramachandran plot was utilised in the PROCHECK tool, and the modelled protein was evaluated and validated (Supplementary Material S1). In the model, 92.7% of residues were found in the most favoured regions (A, B, and L), 6.8% in the additional allowed regions (a, b, l, and p), 0.5% in the generously allowed regions (∼a, ∼b, ∼l, and ∼p), and no residues in the disallowed regions. As a result, the percentage distribution of amino acid residues revealed that the predicted nitrilase structure is of high quality.

### Molecular dynamic simulation

**FIGURE 7:**
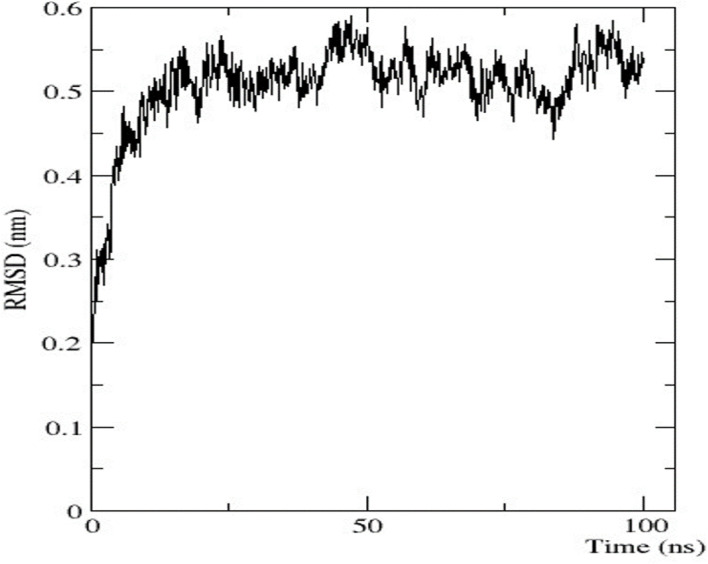
Graphical representation of RMSD.

To analyse the flexibility and stability of the best-predicted protein structure provided by AlphaFold2, a time-dependent molecular dynamic simulation was conducted at 100 ns employing the GROMACS forcefield on the WebGRO server. The RMSD value of the nitrilase protein structure showed equilibrium with an average of 0.2 nm. The largest oscillation in RMSD was observed in the 0–10 ns range. Afterwards, the 10-ns RMSD value was stabilised up to 100 ns, with an average value of 0.5 nm (Figure 7). The RMSF value was then assessed to analyse the structural flexibility of the atoms in the backbone of the proteins. The obtained data showed that there were fluctuations in the residues present in the loops of the protein structure (RMSF ≤ 0.5 nm), and this indicates that the complex was flexible in these loop regions (Supplementary Material S2). The conformational changes in the loop structure caused by the flexibility have no impact on the protein structure (Li et al., 2011).

### Protein and ligand preparation

The predicted nitrilase protein and the retrieved crystal structures of 3WUY (Zhang et al., 2014), 3IW3, and 3IVZ (Raczynska et al., 2011) were used to check the binding scores individually by molecular docking analysis and compared. The three structures were determined by X-ray diffraction analysis. The protein structure (3WUY), which was identified from *Synechocystis* sp. PCC 6803 substr. Kazusa, comprises 349 amino acids and has a resolution of 3.10 Å. The other two protein structures, 3IW3 and 3IVZ, were identified in *Pyrococcus abyssi* GE5 and have a length of 262 amino acid sequences with resolutions of 1.80 Å and 1.57 Å, respectively. For the protein–ligand interaction study, nitriles such as acrylonitrile, benzonitrile, dichlobenil, fumaronitrile, malanonitrile, and succinonitrile were retrieved. Proteins and ligand molecules were converted to the PDBQT format and were ready for docking analysis.

### Active site predictions

The CASTp server identified the active site for the predicted nitrilase protein and the crystal structure of the proteins. The surface area measurement and cavity volumes were predicted. For the predicted protein, the area of the active site was 677.110 Å2, and the volume was 645.046 Å3. The area of the active site for 3WUY, 3IW3, and 3IVZ was 983.426 Å2, 61.447 Å2, and 188.905 Å2 and the volume was 1,129.287 Å3, 31.456 Å3, and 60.553 Å3, respectively. All the pockets chosen for the proteins have a high surface area and volume for the specific enzyme-binding site (Barman et al., 2020).

### Molecular docking analysis

Using AutoDockTools-1.5.7, a grid file was generated for all the proteins. When the model overlapped with the template, an acceptable range of RMSD was determined to be 2.0, which is regarded as satisfactory docking (Castro-Alvarez et al., 2017). The docking analysis was completed using AutoDock Vina, as tabulated in Table 1. The docking results produced nine ligand-binding poses, of which the one with the lowest RMSD (value 0) was selected, indicating a true binding pose. The protein–ligand interaction showed that these proteins’ docking scores are almost similar when they bind to six different ligands.

**TABLE 1:** Docking scores of proteins binding with ligands.

Nitrile	Predicted nitrilase	3WUY	3IW3	3IVZ
Acrylonitrile	−2.9	−3.2	−2.9	−3.5
Benzonitrile	−4.9	−5.4	−5.0	−4.7
Dichlobenil	−5.4	−5.9	−5.2	−4.9
Fumaronitrile	−3.8	−3.6	−3.6	−3.3
Malononitrile	−3.2	−3.4	−3.0	−3.1
Succinonitrile	−3.6	−3.9	−3.4	−3.4

**FIGURE 8:**
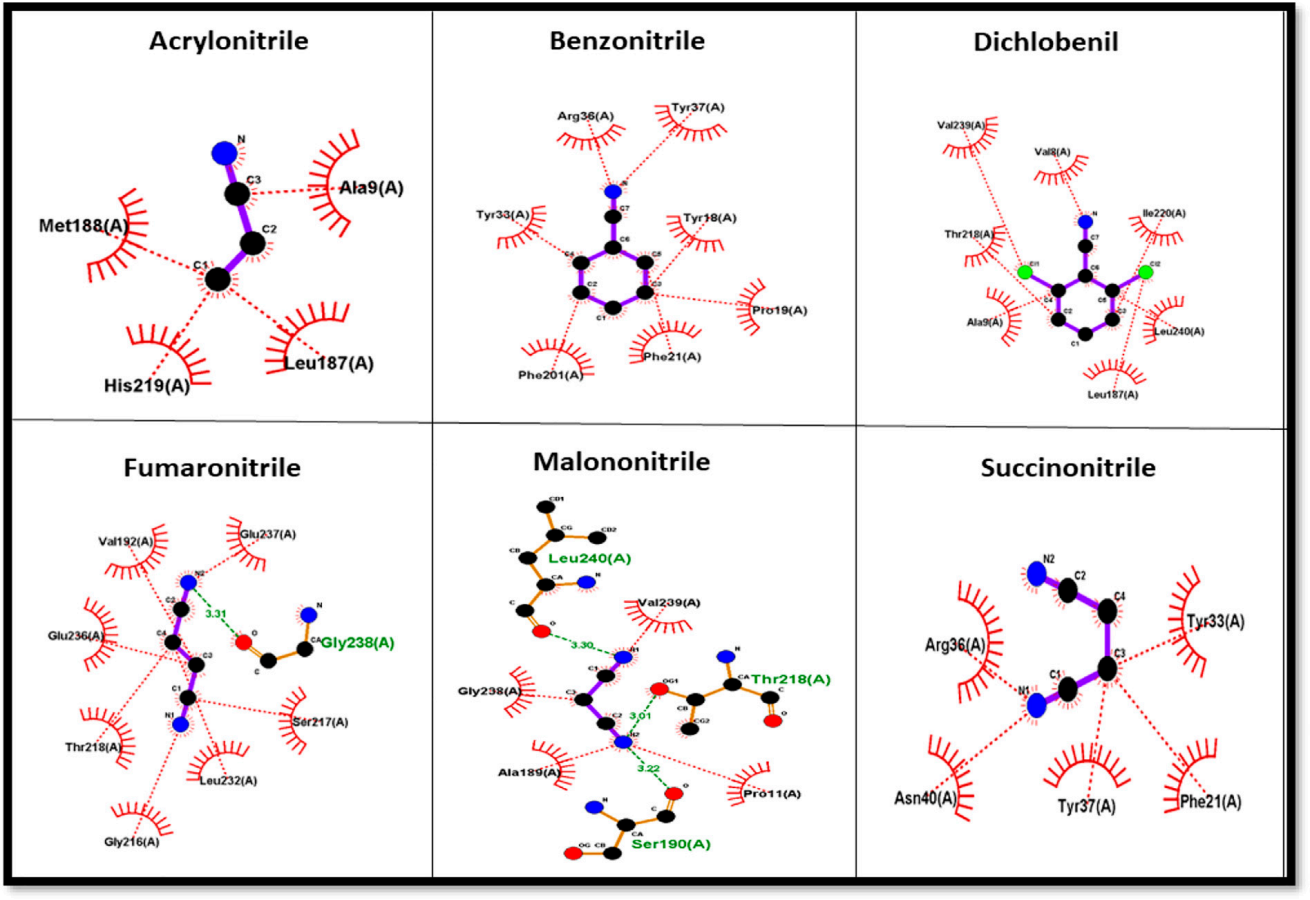
Amino acid interaction with nitrile ligands.

The protein–ligand complex of the predicted nitrilase protein was analysed to determine the nature of the interactions between the amino acids and the ligands using LigPlot + (Figure 8).

Nitrile (C≡N) conversion involves a nucleophilic attack on the electrophilic carbon (electrophilic) by the nucleophilic group present in the side chain of amino acids in the active site, resulting in the formation of carboxyl groups (COOH). Cysteine, serine, threonine, tyrosine, glutamic acid, aspartic acid, lysine, arginine, and histidine have nucleophilic R groups and can act as nucleophilic donors. Arginine and tyrosine amino acids serve as functional converters in benzonitrile and succinonitrile. Threonine and glutamic acid play a functional role in the catalysis of fumaronitrile and serine in malononitrile. The nucleophilic attack on acrylonitrile and dichlobenil is mediated by histidine and threonine, respectively.

## Discussion

Microbial nitrilase, an enzyme of the nitrilase superfamily, is a great option for numerous industrial applications and bioremediation procedures. Functional metagenomics enabled the identification of the novel nitrilase enzyme from environmental sources, which represent a non-culturable source of the enzyme. With the help of artificial intelligence and machine learning in metagenomics, novel enzyme candidates can be identified for potential use in bioremediation and therapeutics. Ligand molecules are bound to this active site of the protein, and the identification of their protein–ligand binding efficacy will lead to drug discovery, which is beneficial for the advancement of green chemistry.

## Data Availability

The data presented in the study are deposited in the NCBI SRA repository with the link http://www.ncbi.nlm.nih.gov/bioproject/941822 and accession number - PRJNA941822.

## References

[B1] Amrutha M. Nampoothiri K. M. (2022). *In silico* analysis of nitrilase-3 protein from Corynebacterium glutamicum for bioremediation of nitrile herbicides. J. Genet. Eng. Biotechnol. 20 (1), 51. 10.1186/s43141-022-00332-5 35348933 PMC8964915

[B2] Andrews S. (2010). FastQC: A quality control tool for high throughput sequence data. Cambridge, United Kingdom: Babraham Bioinformatics, Babraham Institute.

[B3] Barman U. D. Saha S. K. Kader M. A. Jamal M. A. H. M. Sharma S. P. Samad A. (2020). Clinicopathological and prognostic significance of GPC3 in human breast cancer and its 3D structure prediction. Netw. Model. Anal. Heal. Inf. Bioinforma. 9 (1), 24. 10.1007/s13721-020-00234-x

[B4] Bekker H. Berendsen H. J. C. Van Drunen R. Van Der Spoel D. (1993). Gromacs: A parallel computer for molecular dynamics simulations. SINGAPORE: World Scientific Publishing.

[B5] Bin Kwong Q. Ong A. L. Tammi M. (2017). Inspection of sequence quality. Bioinformatics 2017, 49–66. 10.1142/9789813144750_0003

[B6] BIOCATALYSIS OF DIFFERENT NITRILES TO VALUABLE ACIDS (2021). “Smally sinha and vinod kumar nigam,” in Department of bio-engineering (Mesra, Ranch: Birla Institute of Technology), 2662–2669. 10.13040/IJPSR.0975-8232.12(5).2662-69

[B7] Brunner S. Eppinger E. Fischer S. Gröning J. Stolz A. (2018). Conversion of aliphatic nitriles by the arylacetonitrilase from Pseudomonas fluorescens EBC191. World J. Microbiol. Biotechnol. 34 (7), 91. 10.1007/s11274-018-2477-9 29896645

[B8] Burley S. K. Berman H. M. Kleywegt G. J. Markley J. L. Nakamura H. Velankar S. (2017). Protein Data Bank (PDB): The single global macromolecular structure archive. Methods Mol. Biol. 1607, 627–641. 10.1007/978-1-4939-7000-1_26 28573592 PMC5823500

[B9] Castro-Alvarez A. Costa A. M. Vilarrasa J. (2017). The performance of several docking programs at reproducing protein-macrolide-like crystal structures. Molecules 22, 136. 10.3390/molecules22010136 28106755 PMC6155922

[B10] Eberhardt J. Santos-Martins D. Tillack A. F. Forli S. (2021). AutoDock Vina 1.2.0: New docking methods, expanded force field, and Python bindings. J. Chem. Inf. Model. 61 (8), 3891–3898. 10.1021/acs.jcim.1c00203 34278794 PMC10683950

[B11] Ewels P. Magnusson M. Lundin S. Käller M. (2016). MultiQC: Summarize analysis results for multiple tools and samples in a single report. Bioinformatics 32 (19), 3047–3048. 10.1093/bioinformatics/btw354 27312411 PMC5039924

[B12] Gasteiger B. A. Hoogland C. Gattiker A. Duvaud S. Wilkins M. R. Appel R. D. (2005). “Protein identification and analysis tools on the ExPASy server,” in Proteomics protoc. Handbook (Humana Press), 571–667.

[B13] Gasteiger E. Gattiker A. Hoogland C. Ivanyi I. Appel R. D. Bairoch A. (2003). ExPASy: The proteomics server for in-depth protein knowledge and analysis. Nucleic Acids Res. 31 (13), 3784–3788. 10.1093/nar/gkg563 12824418 PMC168970

[B14] Goddard T. D. Huang C. C. Meng E. C. Pettersen E. F. Couch G. S. Morris J. H. (2018). UCSF ChimeraX: Meeting modern challenges in visualization and analysis. Protein Sci. 27 (1), 14–25. 10.1002/pro.3235 28710774 PMC5734306

[B15] Gong J. S. Lu Z. M. Li H. Shi J. S. Zhou Z. M. Xu Z. H. (2012). Nitrilases in nitrile biocatalysis: Recent progress and forthcoming research. Microb. Cell. Fact. 11, 142–218. 10.1186/1475-2859-11-142 23106943 PMC3537687

[B16] Gurevich A. Saveliev V. Vyahhi N. Tesler G. (2013). Quast: Quality assessment tool for genome assemblies. Bioinformatics 29 (8), 1072–1075. 10.1093/bioinformatics/btt086 23422339 PMC3624806

[B17] Høie M. H. Kiehl E. N. Petersen B. Nielsen M. Winther O. Nielsen H. (2022). NetSurfP-3.0: Accurate and fast prediction of protein structural features by protein language models and deep learning. Nucleic Acids Res. 50 (W1), W510–W515. 10.1093/nar/gkac439 35648435 PMC9252760

[B18] Johnson M. Zaretskaya I. Raytselis Y. Merezhuk Y. McGinnis S. Madden T. L. (2008). NCBI blast: A better web interface. Nucleic Acids Res. 36, W5–W9. 10.1093/nar/gkn201 18440982 PMC2447716

[B19] Jumper J. Evans R. Pritzel A. Green T. Figurnov M. Ronneberger O. (2021). Highly accurate protein structure prediction with AlphaFold. Nature 596 (7873), 583–589. 10.1038/s41586-021-03819-2 34265844 PMC8371605

[B20] Kim S. Chen J. Cheng T. Gindulyte A. He J. He S. (2020). PubChem in 2021: New data content and improved web interfaces. Nucleic Acids Res. 49 (D1), D1388–D1395. 10.1093/nar/gkaa971 PMC7778930 33151290

[B21] Klasberg S. Bitard-Feildel T. Mallet L. (2016). Computational identification of novel genes: Current and future perspectives. Bioinform. Biol. Insights 10, 121–131. 10.4137/BBI.S39950 27493475 PMC4970615

[B22] Kupke F. Herz C. Hanschen F. S. Platz S. Odongo G. A. Helmig S. (2016). Cytotoxic and genotoxic potential of food-borne nitriles in a liver *in vitro* model. Sci. Rep. 6, 37631–37711. 10.1038/srep37631 27883018 PMC5121622

[B23] Laskowski R. A. MacArthur M. W. Moss D. S. Thornton J. M. (1993). Procheck: A program to check the stereochemical quality of protein structures. J. Appl. Crystallogr. 26 (2), 283–291. 10.1107/S0021889892009944

[B24] Laskowski R. A. Swindells M. B. (2011). LigPlot+: Multiple ligand-protein interaction diagrams for drug discovery. J. Chem. Inf. Model. 51 (10), 2778–2786. 10.1021/ci200227u 21919503

[B25] Jones L. B. Wang X. Gullapalli J. S. Kunz D. A. (2020). Characterization of the Nit6803 nitrilase homolog from the cyanotroph Pseudomonas fluorescens NCIMB 11764. Biochem. Biophys. Rep. 25, 100893. 10.1016/j.bbrep.2020.100893 PMC7815647 33506113

[B26] Li C. Li Y. Cheng X. Feng L. Xi C. Zhang Y. (2013). Immobilization of Rhodococcus rhodochrous BX2 (an acetonitrile-degrading bacterium) with biofilm-forming bacteria for wastewater treatment. Bioresour. Technol. 131, 390–396. 10.1016/j.biortech.2012.12.140 23376196

[B27] Li D. Liu C. M. Luo R. Sadakane K. Lam T. W. (2015). Megahit: An ultra-fast single-node solution for large and complex metagenomics assembly via succinct de Bruijn graph. Bioinformatics 31 (10), 1674–1676. 10.1093/bioinformatics/btv033 25609793

[B28] Li M. H. Luo Q. Xue X. G. Li Z. S. (2011). Molecular dynamics studies of the 3D structure and planar ligand binding of a quadruplex dimer. J. Mol. Model. 17 (3), 515–526. 10.1007/s00894-010-0746-0 20508957

[B29] Marchler-Bauer A. Bo Y. Han L. He J. Lanczycki C. J. Lu S. (2017). CDD/SPARCLE: Functional classification of proteins via subfamily domain architectures. Nucleic Acids Res. 45 (D1), D200–D203. 10.1093/nar/gkw1129 27899674 PMC5210587

[B30] Nigam V. K. Arfi T. Kumar V. Shukla P. (2017). Bioengineering of nitrilases towards its use as green catalyst: Applications and perspectives. Indian J. Microbiol. 57 (2), 131–138. 10.1007/s12088-017-0645-5 28611489 PMC5446835

[B31] Pettersen E. F. Goddard T. D. Huang C. C. Meng E. C. Couch G. S. Croll T. I. (2021). UCSF ChimeraX: Structure visualization for researchers, educators, and developers. Protein Sci. 30 (1), 70–82. 10.1002/pro.3943 32881101 PMC7737788

[B32] Quevillon E. SilVentoinen V. Pillai S. Harte N. Mulder N. ApweileR R. (2005). InterProScan: Protein domains identifier. Nucleic Acids Res. 33, W116–W120. 10.1093/nar/gki442 15980438 PMC1160203

[B33] Raczynska J. E. Vorgias C. E. Antranikian G. Rypniewski W. (2011). Crystallographic analysis of a thermoactive nitrilase. J. Struct. Biol. 173 (2), 294–302. 10.1016/j.jsb.2010.11.017 21095228

[B34] Egelkamp R. Zimmermann T. Schneider D. Hertel R. Daniel R. (2019). Impact of nitriles on bacterial communities. Front. Environ. Sci. 7, 1–14. 10.3389/fenvs.2019.00103

[B35] Schrödinger L. (2000). The PyMOL molecular graphics system. Version 1.2r3pre.

[B36] Sewell B. T. Berman M. N. Meyers P. R. Jandhyala D. Benedik M. J. (2003). The cyanide degrading nitrilase from Pseudomonas stutzeri AK61 is a two-fold symmetric, 14-subunit spiral. Structure 11 (11), 1413–1422. 10.1016/j.str.2003.10.005 14604531

[B37] Tamames J. Puente-Sánchez F. (2019). SqueezeMeta, A highly portable, fully automatic metagenomic analysis pipeline. Front. Microbiol. 9, 3349. 10.3389/fmicb.2018.03349 30733714 PMC6353838

[B38] Tamura K. Stecher G. Kumar S. (2021). MEGA11: Molecular evolutionary genetics analysis version 11. Mol. Biol. Evol. 38 (7), 3022–3027. 10.1093/molbev/msab120 33892491 PMC8233496

[B39] Tanii H. (2017). Allyl nitrile: Toxicity and health effects. J. Occup. Health 59 (2), 104–111. 10.1539/joh.16-0147-RA 28132970 PMC5478528

[B40] Tian W. Chen C. Lei X. Zhao J. Liang J. (2018). CASTp 3.0: Computed atlas of surface topography of proteins. Nucleic Acids Res. 46 (W1), W363–W367. 10.1093/nar/gky473 29860391 PMC6031066

[B41] Veselá A. B. Franc M. Pelantova H. Kubac D. VejVoda V. Sulc M. (2010). Hydrolysis of benzonitrile herbicides by soil actinobacteria and metabolite toxicity. Biodegradation 21 (5), 761–770. 10.1007/s10532-010-9341-4 20204468

[B42] Wang M. X. (2015). Enantioselective biotransformations of nitriles in organic synthesis. Acc. Chem. Res. 48 (3), 602–611. 10.1021/ar500406s 25699471

[B43] Zhang L. Yin B. Wang C. Jiang S. Wang H. Yuan Y. A. (2014). Structural insights into enzymatic activity and substrate specificity determination by a single amino acid in nitrilase from Syechocystis sp. PCC6803. J. Struct. Biol. 188 (2), 93–101. 10.1016/j.jsb.2014.10.003 25450592

