# Supplementary figures and images for: Functional metagenomics uncovers nitrile-hydrolysing enzymes in a coal metagenome
Source: Front Mol Biosci. 2023 Mar 17;10:1123902. doi: 10.3389/fmolb.2023.1123902 (PMC10063848; doi:10.3389/fmolb.2023.1123902)

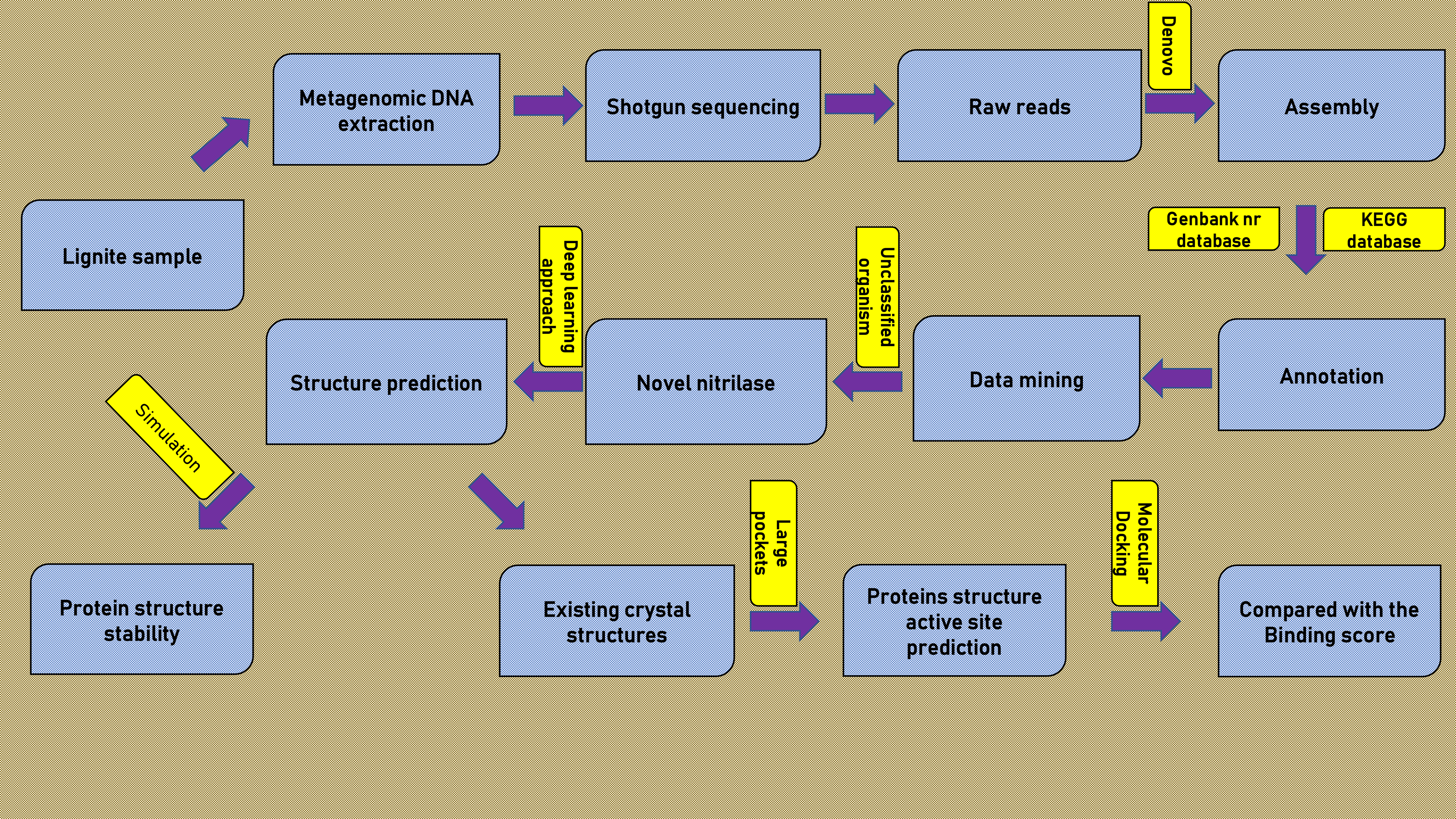

Supplement: Supplementary file 1 [file Image3.TIFF]

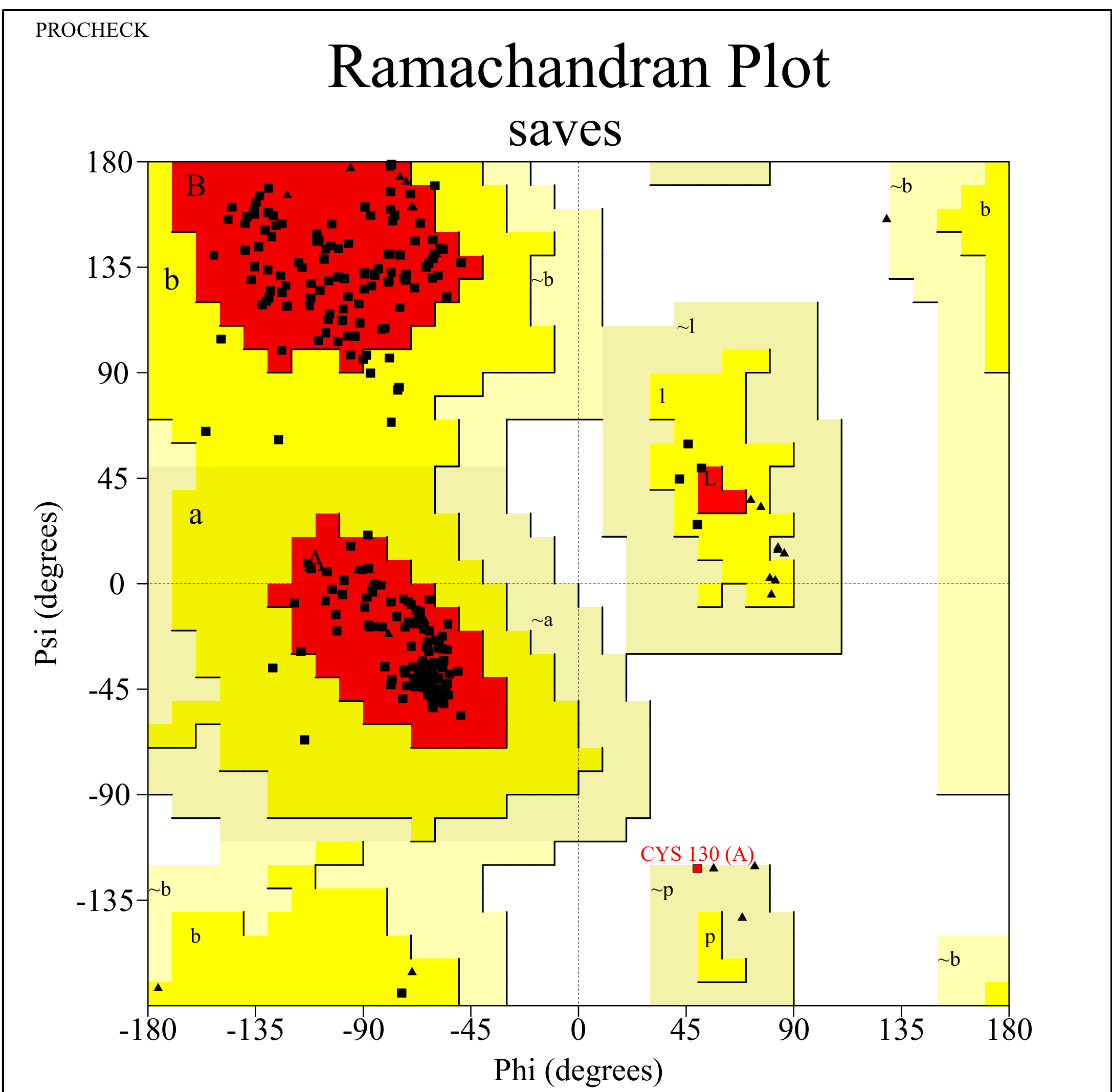

Supplement: Supplementary file 2 [file Image1.TIFF]
